# The Potential of Plant-Derived Foods to Treat Glaucoma: A Review

**DOI:** 10.3390/nu18071080

**Published:** 2026-03-27

**Authors:** Jinze Liu, Zhongmei He

**Affiliations:** College of Chinese Medicinal Materials, Jilin Agricultural University, Changchun 130118, China; liujinze0602@126.com

**Keywords:** glaucoma, food-derived, plants, bioactive components, intraocular pressure

## Abstract

Glaucoma, characterized by progressive retinal ganglion cell degeneration and optic nerve damage, is the leading cause of irreversible blindness worldwide. Multiple risk factors influence the pathogenesis and progression of glaucoma. Food-derived bioactive components have emerged as a new area of interest to overcome the limitations of current standard treatments due to their antioxidant and anti-inflammatory activities and multi-target mechanisms. In this context, various plant-derived foods, such as *Lycium barbarum*, *Ganoderma lucidum*, *Cryptotanshinone*, *Scutellaria baicalensis*, *Silybum marianum*, *Astragalus membranaceus*, *Ginkgo biloba*, *Panax ginseng*, *Crocus sativus*, and resveratrol, have shown potential mechanisms for treating glaucoma. These bioactive components may address oxidative damage, neuroinflammation, and elevated intraocular pressure, which may be due to the modulation of multiple signaling pathways, including JAK2/STAT3, PI3K/AKT, MEK/ERK/CREB, cAMP/PKA/CREB, and others. However, further clinical trials are needed to validate dosage, bioavailability, and long-term safety. This review highlights the potential of bioactive components from plant-derived foods, offering a reference for further investigation into their effects on glaucoma.

## 1. Introduction

**Figure 1:**
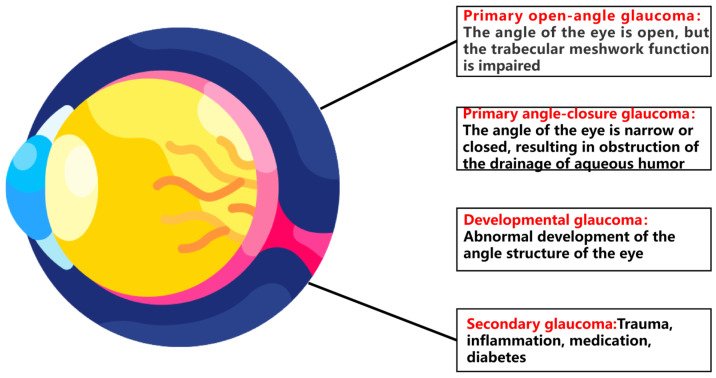
Main classifications of glaucoma. It illustrates four major types of glaucoma, namely primary open-angle glaucoma, primary angle-closure glaucoma, developmental glaucoma, and secondary glaucoma.

**Figure 2:**
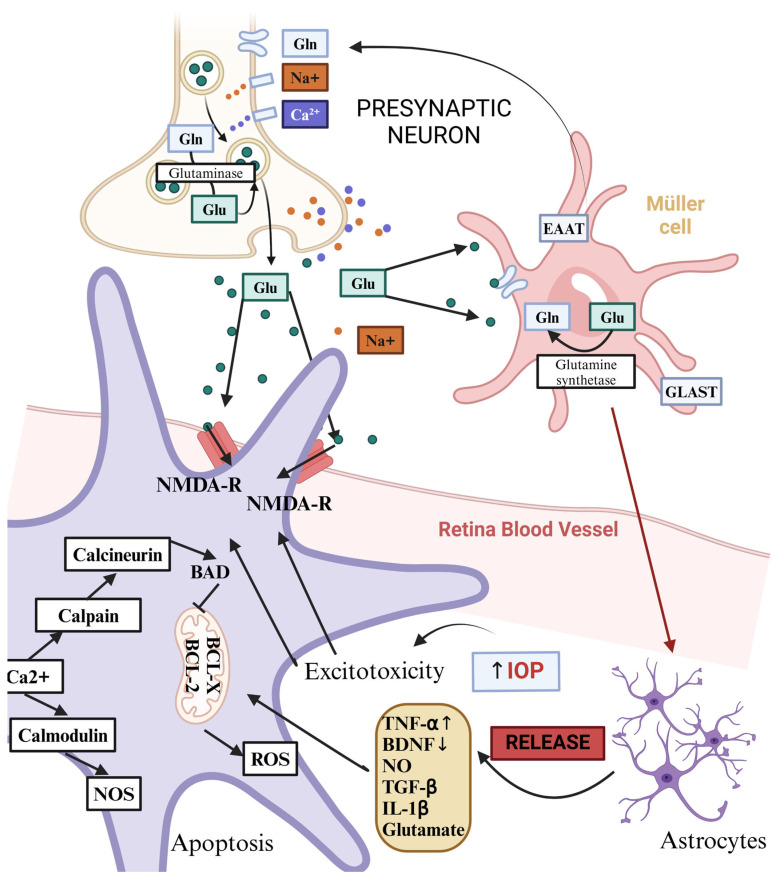
Pathogenesis of glaucoma. Image created with BioRender.com, with permission. The circulation of glutamate between neurons and Müller cells; calcium influx mediated by NMDA receptors, mitochondrial dysfunction, and downstream apoptotic signaling pathways.

Glaucoma is an insidious, irreversible chronic eye disease that is closely associated with elevated intraocular pressure (IOP) and is the second most common cause of blindness worldwide after cataracts [1]. It is characterized by progressive optic neuropathy caused by retinal ganglion cell (RGC) injury, visual field loss and vision loss, and optic nerve damage, resulting in progressive vision loss [2]. According to different etiology, pathology, and clinical manifestations, glaucoma can be divided into three categories, primary glaucoma, secondary glaucoma, and congenital glaucoma (Figure 1) [3]. Of these, primary glaucoma is the most common and includes primary open-angle glaucoma (POAG) and primary angle-closure glaucoma (PACG). According to epidemiological data, the number of glaucoma patients will increase to 111.8 million worldwide by 2040 [4]. The pathogenesis involves elevated IOP, optic nerve damage, oxidative stress, and inflammatory response [5] (Figure 2).

Elevated IOP can lead to optic nerve damage. Increased sieve plate pressure from the optic nerve reduces the transmission of trophic signals (brain-derived neurotrophic factor (BNDF)) and nerve growth factor (NGF) from the terminal axon to the neuronal cell body, as well as the transport of nutrients and oxygen to the layers of the retina that produce hypoxia and ischemia. The RGC axon is highly active and energy-dependent, which correlates with the large number of mitochondria in this segment of the optic nerve. Changes in energy supply result in altered axoplasmic flow from the lateral geniculate nucleus and superior colliculus to RGCs, helping to reduce neurotrophic factors and axonal damage [6]. Müller cells can be neuroprotective after injury due to the release of neurotrophic factors (e.g., BDNF) but, depending on the time course of the injury, they can be involved in inflammatory processes [7]. Currently, the main treatments for glaucoma include IOP-lowering medications, laser therapy, and surgery [8]. However, these treatments do not completely stop the progression of the disease and have certain side effects and risks [9]. This has led to an increasing interest in treatment avenues that have fewer side effects and are safer.

In recent years, as research on foodborne bioactive components has deepened, an increasing number of dietary source plants and their active substances have been identified for their potential value in glaucoma treatment. These diet-derived bioactive components offer novel insights for the potential anti-glaucoma effects shown in preclinical studies through their unique antioxidant–anti-inflammatory synergies, IOP regulation mechanisms, and neuroprotective effects. Compared with traditional medicinal plants, foodborne active substances demonstrate distinct advantages in terms of bioavailability and long-term safety due to their natural food substrate properties [10]. Based on the “food and medicine sharing the same origin” theory, many traditional medicinal plants have shown great potential in the development of modern functional foods. The examples of medicinal plants cited in this article are precisely based on this cutting-edge research progress. This paper systematically examines the relationship between the pathological mechanisms of glaucoma and factors such as oxidative stress and chronic inflammation. It further analyzes the limitations of existing treatment methods. The regulatory effects of ten representative plant-derived foods (e.g., *Lycium barbarum*, which is rich in polysaccharides) and their active components on the progression of glaucoma are reviewed, and their mechanisms are discussed from the perspective of reducing intraocular pressure and protecting RGCs. Finally, this review evaluates recent advancements in the potential benefits and limitations of foodborne bioactive components, focusing on their potential anti-glaucoma effects shown in preclinical studies, to promote their application.

## 2. Methods

**Figure 3:**
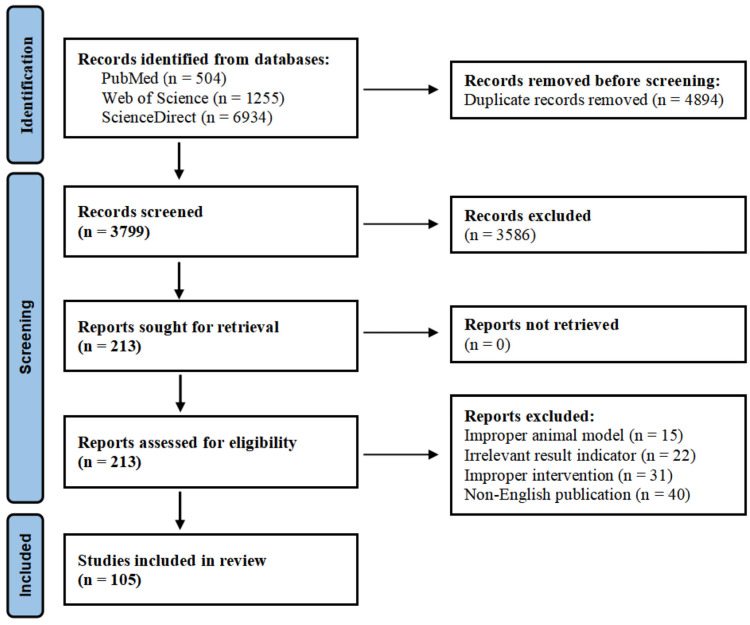
PRISMA flowchart of the literature screening process.

A comprehensive literature review was conducted to determine the investigation into the potential of foodborne bioactive components with therapeutic potential against glaucoma research. The databases utilized included PubMed, Web of Science, and ScienceDirect (as of March 2025). We searched for relevant articles using the keywords glaucoma, foodborne bioactive components, intraocular pressure, intraocular pressure, neuroprotection, and retinal ganglion cells. In this paper, we synthesize the results of studies that have elucidated the pathogenesis of glaucoma and explored the potential therapeutic effects and molecular mechanisms of foodborne bioactive components. Our goal is to provide researchers with a comprehensive overview of recent preclinical advances in the use of phytochemical food factors for glaucoma management and to offer insights into potential therapeutic approaches based on these findings. This review was written according to PRISMA criteria [11], and the literature screening process is shown in Figure 3. The literature screening process of this systematic review followed the established exclusion criteria to control the risk of bias. Specifically, ① inappropriate animal models refer to those using non-standard species that do not conform to the pathological and physiological mechanisms of human diseases, or models that have not been successfully induced; ② the irrelevant result indicator only detects biochemical or inflammatory markers but does not report the core outcome indicators; and ③ inappropriate intervention measures include drug dosages exceeding clinical levels. These three types of literature were all excluded at the full-text screening stage (as shown in Figure 3) to ensure the reproducibility of the data and its clinical reference value.

## 3. Pathogenesis of Glaucoma in Modern Medicine

Glaucoma, characterized by progressive retinal ganglion cell (RGC) apoptosis and optic nerve atrophy, is a group of ocular diseases that severely affect visual function; glaucoma has been a hot and difficult topic of ophthalmic medical research. Its pathogenesis is complex and involves multiple levels of physiopathological processes [12], genetic factors, and environmental factors [13]. The following article focuses on the relationship between physiopathological processes and glaucoma, including elevated IOP, optic nerve damage [14], oxidative stress, and inflammatory response [15], with a view to providing new ideas with therapeutic potential against glaucoma.

### 3.1. Elevated IOP

**Figure 4:**
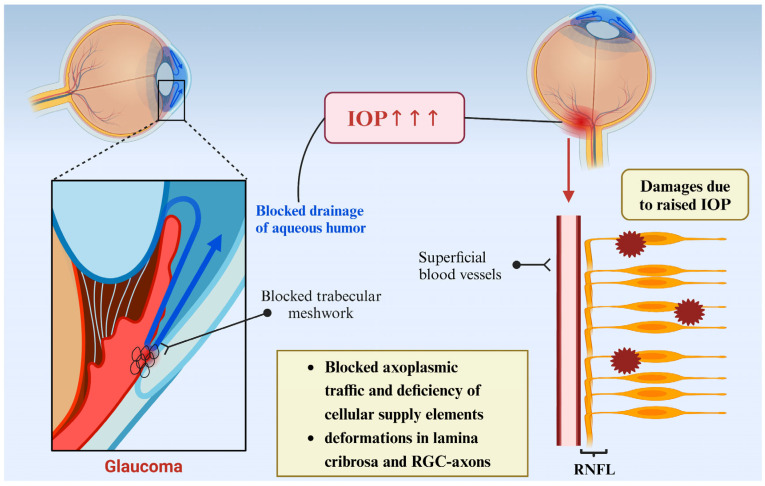
RGC denaturation due to elevated IOP. Image created with BioRender.com, with permission. The pathological process includes obstruction of aqueous humor outflow, blockage of the trabecular meshwork, retardation of axoplasmic flow, deformation of the optic nerve head, and damage to the axons of retinal ganglion cells.

IOP, as the name suggests, refers to the pressure exerted by the contents of the eyeball on the lining of the eye [16]. It is the result of the interaction between the circulation of fluid (mainly aqueous humor) inside the eyeball and the elasticity of the eyeball wall [17]. Aqueous humor (AH) is produced by the ciliary body; it passes through the pupil into the anterior chamber (AC) and then exits the eye through structures such as the trabecular meshwork (TM) and the scleral venous sinus, maintaining the dynamic balance of IOP [18]. Under normal circumstances, adult intraocular pressure is maintained in the range of 5~21 mmHg [19], which is an important physiological indicator inside the eyeball, and its dynamic balance is crucial to maintain the normal structure and function of the eyeball. When the intraocular pressure exceeds the normal range, especially when it continues to rise, it may have adverse effects on the optic nerve of the eye and then lead to a series of eye diseases, among which the most well-known is glaucoma [20]. Elevated IOP is a direct cause of glaucoma pathogenesis and a major modifiable risk factor for glaucoma [21]. The causes of elevated IOP in glaucoma are varied, with the most widely studied being closely related to an imbalance in the atrial aqueous circulation [22]. Obstruction of aqueous drainage leads to impaired transport of substances within the axon and deformation of the RGC axon, which in turn leads to increased intraocular pressure and damage (Figure 4).

**Figure 5:**
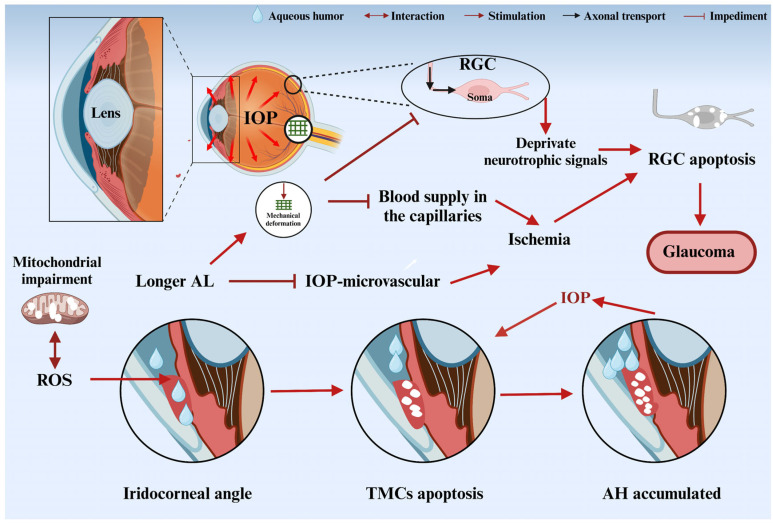
The important role of oxidative stress in glaucoma. Image created with BioRender.com, with permission. Mechanical compression leads to deprivation of neurotrophic factors and ischemia, resulting in apoptosis of trabecular meshwork cells and accumulation of aqueous humor.

When IOP exceeds the tolerance threshold of the optic nerve, apoptosis of TMCs is induced by oxidative stress, and mitochondrial dysfunction [23] leads to elevated IOP (Figure 5). Elevated IOP further leads to cell death of TMCs [24]. The elevated IOP acts directly on Lamina Cribrosa (LC), leading to mechanical distortion of optic nerve fibers and disruption of axoplasmic flow [25]. Abnormal deposition of LC extracellular matrix (ECM) (e.g., collagen, elastin) and imbalanced activity of metalloproteinases (MMPs) also lead to LC sclerosis [26], further limiting axonal compliance. Axonal compression leads to the inability of neurotrophic factors (e.g., BDNF, CNTF) to be retrogradely transported from target tissues (e.g., lateral geniculate body) to the cytosol of RGCs [27], triggering neurotrophic deprivation apoptosis and a decrease in autophagic clearance. It further accelerates neuronal degeneration and aggravates the process of glaucoma, accompanied by concomitant systemic diseases, such as diabetes mellitus [28] and hypertension [29]. In addition, longer axial length (AL) in myopic patients disrupts intraocular microvascular autoregulation and exacerbates mechanical deformation of the LC, making eyes with a longer AL more susceptible to IOP-induced reductions in blood flow, leading to apoptosis of RGCs due to ischemia [30]. These factors interact to affect aqueous effluent discharge, further exacerbating IOP elevation and glaucoma progression. Even when IOP is normalized (normal tonic glaucoma, NTG), RGCs may still be impaired [12]. It was shown that pathophysiological changes in the retina and RGCs were observed after IOP normalization in a recovery model of elevated IOP in the anterior chamber of perfused mice. Continued loss of RGCs after IOP normalization was found to persistently cause vision loss [31]. Nam et al. [32] investigated the effects of the anti-apoptotic peptides peptain-1 and peptain-3a on the death of primary RGCs in rats and RGCs in vitro in mice with hypertension, demonstrating that the progression of glaucoma can be blocked in an animal model of glaucoma by inhibiting hypertension-mediated activation of retinal glial cells.

In recent years, glaucoma, as one of the leading causes of irreversible vision loss worldwide, has been a hot topic in ophthalmic research due to its complex pathological mechanisms. Among them, optic nerve damage serves as the core pathological process of glaucoma. In recent years, researchers have been interested in neuroprotective therapy as a cutting-edge therapeutic strategy to treat neurodegenerative diseases by protecting the brain system from harmful events. Nowadays, glaucoma is recognized as a chronic neurodegenerative disease and is one of the main causes of limited vision in the elderly worldwide [33]. For this factor, protecting the optic nerve as a new approach to stop or slow down the progression of morphological and functional glaucomatous damage is in line with the treatment strategies suggested for other neurodegenerative diseases [34]. Elevated IOP also affects the blood supply to the optic nerve, resulting in decreased perfusion pressure in the short posterior ciliary artery, decreased blood flow, and optic nerve ischemia and hypoxia [35]. Optic nerve ischemia and hypoxia can lead to a series of secondary pathological changes, such as glial cell proliferation [36], axon swelling [37] and degeneration of optic nerve, reduced nitric oxide (NO) synthesis [38], increased vasoconstrictor factor ET-1 [39], and microangiopathy. More recently, an increasing amount of research has focused on further mechanisms by which ET-1 regulates RGC death [40,41]. As previously mentioned, JNK signaling mediates neurodegeneration in glaucoma [42]. In addition, ET-1 can induce RGC death and activate c-Jun through the Jun-dependent pathway. It was further demonstrated that ET-1 triggers c-Jun through the endothelin receptor, and c-Jun feedback increases the expression of the endothelin receptor [43]. Further studies are needed to identify upstream regulators and downstream targets of JUN signaling. This evidence may help to elucidate the potential mechanism of endothelin regulating RGC death and explore the protective effect of optic nerve on the development of glaucoma. These pathological changes further exacerbate the damage to the optic nerve, leading to a decrease in its conduction function. In addition, optic nerve ischemia may trigger oxidative stress [44]. This is because intracellular free radical production increases and antioxidant capacity decreases in ischemia, leading to increased cellular damage and exacerbation of glaucoma symptoms. It is worth noting that by reducing IOP to alleviate mechanical compression and ischemia, a survival environment is provided for RGCs. On the other hand, by directly regulating the survival signaling pathways within RGCs, the cell’s anti-injury ability can be enhanced.

### 3.2. Oxidative Stress and Mitochondrial Dysfunction

**Figure 6:**
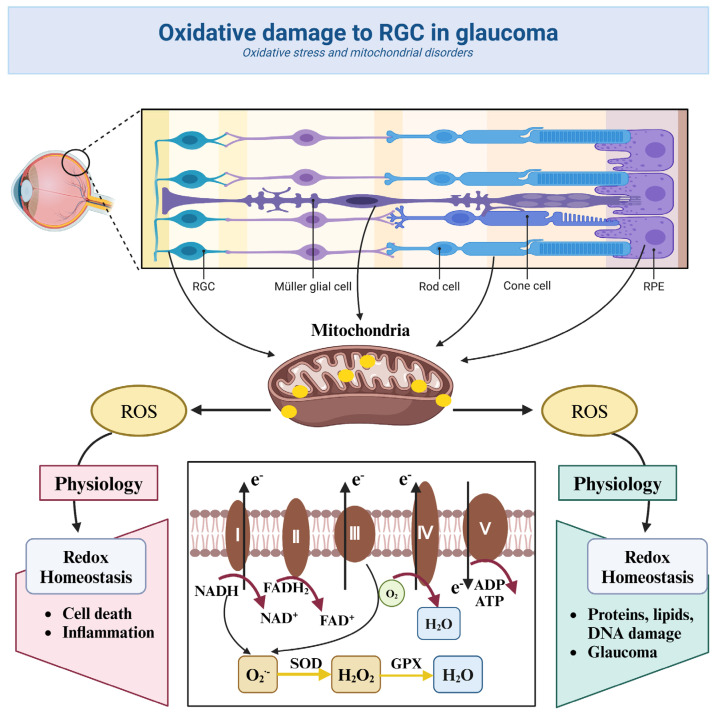
Oxidative damage to RGC in glaucoma. Image created with BioRender.com, with permission. Damage to the various layers of the retina, production of reactive oxygen species (ROS), antioxidant enzyme system, and electron transport chain.

In addition to the above association with glaucoma incidence, oxidative stress levels were inversely associated with increased glaucoma severity [45]. Oxidative stress refers to an imbalance between the production of reactive oxygen species (ROS) in the body and the ability of antioxidants and repair systems [46]. In the body, mitochondria is one of the main sources of endogenous ROS in cells [47]. During the pathogenesis of glaucoma, mitochondrial function may be impaired, resulting in increased ROS production [48]. In addition, enzymes such as xanthine oxidase (XO) and NADPH oxidase NOX2 can catalyze the production of ROS under certain conditions [49]. These enzymes may be more active in glaucoma patients, exacerbating ROS production [50]. Notably, elevated ROS levels can also be caused through complexes I and III, leading to oxidative damage to mitochondrial DNA (mtDNA), resulting in mitochondrial protein and membrane defects [51]. ROS (O^2−^, H_2_O_2_) can also be generated during mitochondrial respiration when high-energy electrons escape before reaching the final receptor O^2^ [52]. Mitochondrial oxidative stress can also lead to mitochondrial dysfunction and highly contribute to extraretinal lesions [53]. In this state, the overproduction of ROS leads to damage to lipid, protein, and DNA structures, which in turn triggers cellular dysfunction and death [54]. Most notably, it can directly affect the normal function of RGCs. ROS can directly attack the cell membranes, proteins, and DNA of RGCs, leading to disruption of cellular structure and function. This direct damaging effect may lead to apoptosis and death of RGCs [55]. ROS accumulation inhibits the antioxidant defense system of the Nrf2/ARE pathway, leading to damage of RGCs [56]. Altered mitochondrial membrane permeability activates the pro-apoptotic protein Bax and inhibits the anti-apoptotic protein Bcl-2, triggering cytochrome C release and caspase cascade reactions [57]. In addition, ROS, as a second messenger, can activate a series of signaling pathways, such as NF-κB [58], MAPK [59], etc. Activation of these signaling pathways further exacerbates the inflammatory response and cell damage, creating a vicious cycle [60]. ROS can also induce activation and infiltration of immune cells in the retina, thereby triggering an inflammatory response [61]. Inflammatory response not only aggravates the injury of RGCs but also promotes the formation of neovascularization and fibrosis [62]. ROS can also affect the energy metabolism and REDOX balance of RGCs by inhibiting the function of the mitochondrial respiratory chain and reducing the efficiency of ATP production [63], leading to abnormal cell function and death (Figure 6).

### 3.3. Immune Response

Glaucoma is a disease that causes damage to the optic nerve and is often associated with increased pressure in the eye [64]. But recent studies have also found that the immune system may play a role, specifically the involvement of T cells [65]. T cells are part of the immune system that normally recognizes and attacks pathogens [66]. But in autoimmune diseases, T cells may mistakenly attack their own tissues [67]. The study notes that autoantibodies found in glaucoma patients may hint at abnormal activation of the immune system [68].

**Figure 7:**
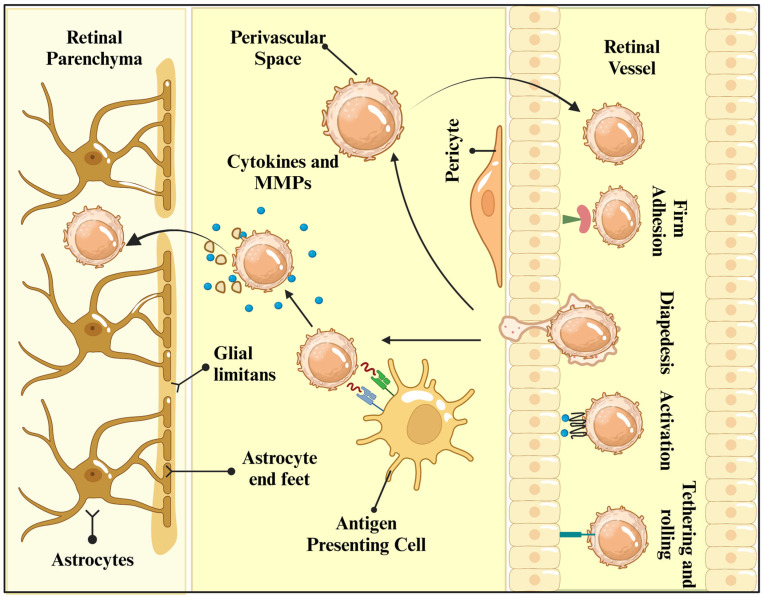
T cell-mediated autoimmunity in glaucoma. Image created with BioRender.com, with permission. The role of cells in immune surveillance.

T cells are attracted to the retina by chemokines (CXCL10, CCL2, etc.), and then they enter the tissue through the blood vessel wall [69]. This process may require adhesion molecules, such as selectin and integrin, to mediate the initial rolling of T cells in the vascular endothelium. Adhesion molecules gradually adhere to, deform, and then cross the blood–retinal barrier (BRB) [70]. In the perivascular space, T cells need to be reactivated by antigen-presenting cells and secrete cytokines and MMP to break through glial cell constraints. This may be due to increased IOP [71] or the secretion of pro-inflammatory factors (TNF-α, IL-6I, and L-1β) [72] and effector T cytokines IFN-γ [73], which activate endothelial cells and microglia and promote the expression of adhesion molecules and chemokines (Figure 7). Thus, in glaucoma, T cell infiltration is the result of a combination of chemokine gradients, adhesion molecule-mediated migration, and autoantigen-driven activation. The initial purpose may be to clear the damage products, but an excessive or dysregulated immune response leads to the death of retinal ganglion cells [74].

### 3.4. Inflammatory Response

**Figure 8:**
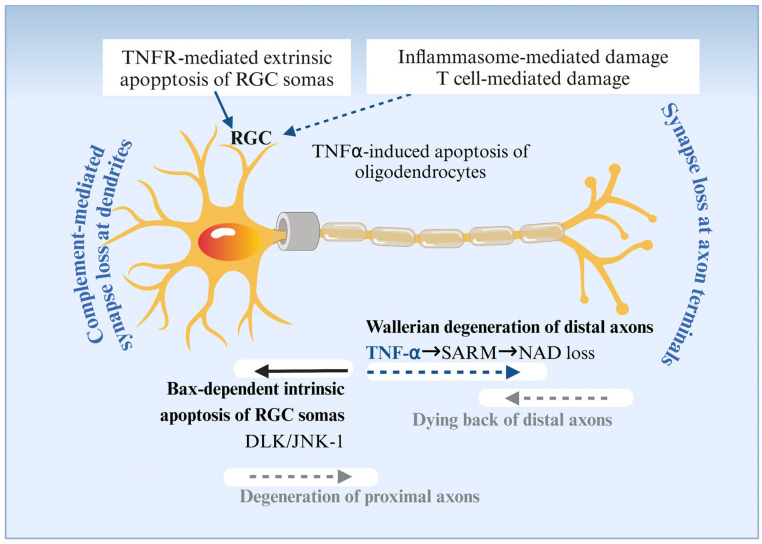
The inflammatory response of RGCs in glaucoma. Image created with BioRender.com, with permission. TNFR-mediated exogenous apoptosis, Bax-dependent endogenous apoptosis, Wallerian degeneration, and damage mediated by inflammasomes/T cells.

Inflammation is the core pathological process of glaucoma [75]. Inflammation is a kind of defense response to injury factors, which is manifested by local tissue deterioration, exudation, and hyperplasia [75]. The glial cells in the retina, Muller cells, astrocytes, and microglia, have a supportive effect on neurons under normal conditions, but they may be activated in pathological states to release inflammatory factors, thus triggering neuroinflammation [76]. TNF is a tumor necrosis factor that not only has considerable pro-inflammatory activity but is also important for the direct toxicity of RGCs in glaucoma [77]. It acts by binding to TNFR (TNF receptor) and can lead to direct RGC apoptosis, glial cell death, and axonal degeneration [78]. This result was corroborated by elevated levels of pro-inflammatory factors detected in the patients’ atrial fluid and blood [79]. TNF has two receptors, TNFR1 and TNFR2 [80], that may mediate different signaling pathways leading to RGC death in different circumstances. In animal models, there is evidence supporting the role of TNF/TNFR in glaucoma [81]. In addition, this has been verified in a number of experiments by knocking out the gene in mice [82]. TNF-α leads to RGC apoptosis through the activation of TNFR, especially TNFR1, which may activate downstream apoptotic pathways, such as the activation of caspase-8 and caspase-3 [83]. TNFα-induced degeneration of RGC axons is mediated by the sterile alpha and TIR motif containing-1 (SARM1) and NAD-dependent pathway [84]. Inflammatory injury to glaucomatous RGCs is illustrated in Figure 8.

### 3.5. Glutamate Excitotoxicity

Glutamate is the main excitatory neurotransmitter in the central nervous system [85]. Under normal conditions, retinal ganglion cells transmit visual signals by releasing glutamate, which binds to specific receptors on downstream neurons to complete the normal process of visual information processing and transmission [86]. Moreover, the excess extracellular glutamate is rapidly taken up and metabolized by the surrounding astrocytes via glutamate transporters, maintaining the extracellular glutamate concentration within a stable and safe range [87]. IOP is an important risk factor in the pathogenesis of glaucoma [88]. Elevated IOP can lead to impaired blood circulation in the retina and optic nerve, resulting in local ischemia and hypoxia [35]. This ischemic and hypoxic state causes depolarization of the nerve endings, prompting the release of large amounts of glutamate from the nerve endings into the extracellular space. At the same time, ischemia and hypoxia also inhibit the activity of glutamate transporters and reduce the uptake capacity of glutamate by astrocytes, resulting in a sharp increase in extracellular glutamate concentration. A high extracellular glutamate concentration will over-activate glutamate receptors on the neuronal cell membrane, and a large number of calcium ions will inwardly flow into the neuronal cell through the receptor channel. Intracellular calcium ion overload activates a series of enzymes such as calpain and phospholipase A2. These enzymes will destroy intracellular proteins, lipids, and other biomolecules, leading to cytoskeletal disintegration, cell membrane damage, etc., and ultimately triggering neuronal cell death [89]. In addition, calcium ions stored in the endoplasmic reticulum are released into the cytoplasm, which also causes an increase in intracellular calcium ion concentration, which in turn triggers neurotoxic effects.

**Figure 9:**
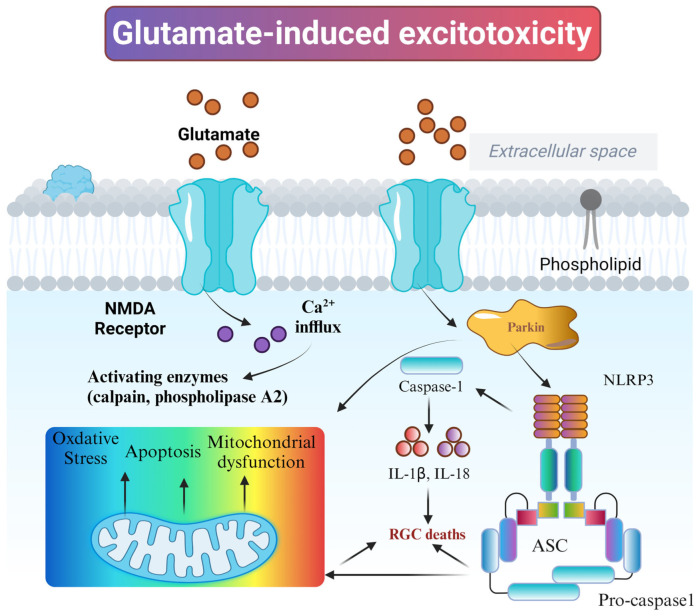
Glutamate-induced excitotoxicity. Image created with BioRender.com, with permission. Activation of NMDA receptors, calcium influx, mitochondrial dysfunction, oxidative stress, and the activation pathway of NLRP3 inflammasome.

In conclusion, glutamate neurotoxicity plays a central role in retinal ganglion cell injury in glaucoma (Figure 9). Excess glutamate over-activates NMDA receptors (NMDARs), as shown in Figure 7; this causes sustained neuronal depolarization and calcium ion (Ca^2+^) in-flow, triggering mitochondrial calcium overload and dysfunction. ROS and Ca^2+^ act as danger signals, directly activating the assembly of inflammatory vesicles by NLRP3. NLRP3 binds to ASC (apoptosis-associated speck-like protein) and pro-caspase-1 and forms inflammatory vesicles that generate active IL-1β and IL-18; it also triggers local inflammation and exacerbates RGC death. This pathway integrates excitotoxicity and inflammatory responses, providing a new direction for neuroprotective therapy in glaucoma.

## 4. Conventional Glaucoma Treatment and Its Limitations

Glaucoma is a kind of eye disease which causes visual field defect and visual acuity decrease due to optic nerve damage caused by the increase in intraocular pressure. Of the several factors that may increase the risk of developing glaucoma, controlling eye pressure is considered the most critical factor with therapeutic potential against glaucoma [90]. Therefore, the primary task with therapeutic potential against glaucoma is to control intraocular pressure. At present, glaucoma is mainly treated by applying drugs directly to the affected area or by taking drugs orally. Then, laser treatment and, if necessary, surgery are performed, each of which has its own advantages and limitations [91] (Table 1).

Eye drops are the most common form of medication used to treat glaucoma [92]. Compared to surgery and laser treatment, medication has a lower risk of direct damage to eye tissue [93]. Most medications have relatively mild side effects under normal use and are suitable for patients who cannot tolerate surgery or laser treatment, such as the elderly or those with multiple systemic diseases. However, glaucoma is a chronic disease, and medication often needs to be continued throughout life. Moreover, some of the drug components may be absorbed into the blood circulation through the mucous membrane of the tear duct, triggering systemic side effects. β-blockers may affect the function of the cardiovascular system and the respiratory system, resulting in slow heart rate, decreased blood pressure, and asthma attacks, and carbonic anhydrase inhibitors may cause metabolic acidosis, hypokalemia, and so on [94,95].

Compared to surgery, the laser treatment process is relatively simple and does not require complex eye incision and tissue suturing. The most typical is a laser peripheral iridotomy [96]. For some specific types of glaucoma, such as primary angle-closure glaucoma in the early stages, laser treatment can effectively relieve pupil block and reduce intraocular pressure, which may contribute to reducing the occurrence of acute attack [97]. The success rate is high, and many patients can be significantly reduced after receiving laser treatment. However, laser therapy has certain limitations; its effect is better mainly in those with early or specific types of glaucoma. For some complex types such as open-angle glaucoma and neovascular glaucoma, the effect of laser treatment may be limited, and it cannot be used as the main treatment [98]. In addition, laser treatment may cause some complications, such as inflammation after laser surgery, acute increase in intraocular pressure, iris bleeding, and so on [99]. Although most of these complications can be treated by drugs and other methods, they still bring some pain and risk to patients.

Surgical treatment can directly change the structure of the eye [100]. New aqueous humor drainage channels have been established to reduce intraocular pressure more effectively [101]. For patients with poor drug and laser treatment, surgery can often achieve better IOP control, providing a strong guarantee for the protection of the optic nerve. After successful surgery, intraocular pressure can be controlled stably for a longer period of time. There is even no need to rely on medication to maintain eye pressure, reducing the hassle and potential side effects of long-term medication. However, there are risks associated with any surgery, and glaucoma surgery is no exception. Surgery can lead to serious complications such as eye infection, bleeding, and tissue damage in the eye, affecting the patient’s vision and eye health [102]. At the same time, it will also cause certain changes to the normal structure of the eye, which may affect other functions of the eye.

Therefore, it is of great significance to find new and effective treatment methods. In recent years, an increasing number of studies have shown that foodborne bioactive components also have some potential in the treatment of eye diseases.

## 5. Potential of Foodborne Bioactive Compounds in Preclinical Anti-Glaucoma Studies

The application of foodborne bioactive compounds derived from plants in glaucoma treatment is mainly based on their multifaceted mechanisms of action, such as antioxidant, anti-inflammatory, and IOP-lowering [103,104]. Oxidative stress is one of the important links in the pathogenesis of glaucoma [105]. Many bioactive compounds have significant antioxidant effects, which can scavenge free radicals and reduce oxidative damage in ocular tissues [106]. Tea polyphenols in green tea and anthocyanins in blueberries have powerful antioxidant properties [107]. These components are able to reduce oxidative stress in ocular tissues, thereby potentially protecting the optic nerve from damage. Inflammatory response is also an important factor in the pathogenesis of glaucoma [108]. Some botanical constituents have anti-inflammatory properties that can inhibit the inflammatory response of ocular tissues and reduce ocular discomfort and damage [109].

Botanical constituents such as curcumin [110] and resveratrol have been shown to have significant anti-inflammatory effects. Elevated IOP is one of the major risk factors for glaucoma. In addition, some bioactive compounds are able to lower IOP by regulating the circulation of atrial fluid in the eye and reducing atrial fluid production. Components such as ginkgolides in Ginkgo biloba leaf extracts have been shown to have IOP-lowering effects [111]. They can improve ocular microcirculation and promote the discharge of atrial fluid, thus lowering IOP and reducing the pressure on the optic nerve. Damage to the optic nerve is the direct cause of vision loss in glaucoma [112]. Some components can protect the optic nerve from damage and promote regeneration and repair of optic nerve cells. Components such as ginsenosides [113] and astragalus polysaccharides [114] have been shown to have significant optic neuroprotective effects. They can improve the survival environment of optic nerve cells and promote the regeneration and repair of nerve cells, thus helping to restore vision. As shown in Table 2, here we summarize studies where bioactive compounds obtained from foodborne compound sources have improved glaucoma by modulating relevant pathways.

### 5.1. Lycium barbarum

As a natural active ingredient, polysaccharide exhibits potential anti-glaucoma effects in preclinical studies [115]. Compared with traditional treatments, polysaccharides have the advantages of fewer side effects and higher safety, and therefore have greater prospects for development and application [116]. In recent years, an increasing number of studies have shown that polysaccharides of foodborne also have some potential in the treatment of ophthalmic diseases [103]. Based on their potential anti-glaucoma effects shown in preclinical studies, the mechanism of action of polysaccharides may involve several aspects [117,118]. Firstly, polysaccharides have an antioxidant effect that scavenges free radicals and reduces damage to ocular tissues caused by oxidative stress. Oxidative stress is one of the important mechanisms in the pathogenesis of glaucoma, and the antioxidant effect of polysaccharides is important for the protection of retinal ganglion cells. Secondly, polysaccharides have anti-inflammatory effects, which can reduce ocular inflammatory reactions and further alleviate glaucoma symptoms. Inflammatory response is also one of the important factors in the development of glaucoma, and the anti-inflammatory effect of polysaccharides helps to reduce the damage and inflammatory response of ocular tissues [119]. In addition, polysaccharides may also play a therapeutic role by regulating the ocular microenvironment and promoting nerve regeneration [120].

*Lycium barbarum* polysaccharide (LBP) is a natural polysaccharide complex extracted from *Lycium barbarum*, which is mainly composed of arabinose, galactose and glucose [121]. Its core pharmacological effects include antioxidant, anti-inflammatory and immunomodulatory [122]. Currently, it has been shown to reduce IOP, protect the normal function of retinal ganglion cells, scavenge free radicals, and reduce ocular inflammatory responses by modulating oxidative stress, further alleviating glaucoma symptoms [123]. It is well known that elevated IOP is the most important factor in glaucomatous optic neuropathy. Therefore, lowering IOP is the most effective and commonly used treatment in glaucoma management [124]. In an acute ocular hypertensive (AOH) mouse model, LBP pretreatment provided relatively good neuroprotection, blocked secondary degeneration, and improved retinal function [125]. The neuroprotective effect of LBP was similarly found in an SD rat model of chronic ocular hypertension, providing proof-of-concept evidence for adjunctive glaucoma therapy [126]. Furthermore, in an in vitro PC12 cell-mimicking glaucoma assay, ethanol extracts of *Lycium barbarum* were found to protect cells from IOP and to be protective against glaucomatous episodes by activating the caveolin-1-dependent pathway through inhibition of matrix metalloproteinase (MMP-9) expression [127]. In addition to the potential anti-glaucoma effects observed in preclinical studies, neuroprotective strategies are necessary to protect healthy neurons and salvage damaged neurons. LBP was also found to protect retinal ganglion cells by lowering mitochondrial membrane potential and reactive oxygen species-induced apoptosis in a 2020 study in a model of oxidative damage to rat RGC-5 cells by CoCl_2_ [128].

### 5.2. Ganoderma lucidum

*Ganoderma lucidum* is a well-known medicinal mushroom whose health benefits have been confirmed by numerous studies [129]. It is widely used as a natural medicine and dietary supplement, especially in China [130]. It possesses more than 400 bioactive compounds including polysaccharides, triterpenoids, polyphenols, sterols, and nucleotides [131]. *Ganoderma lucidum* polysaccharide (GLP), the main extract of *Ganoderma lucidum*, has anti-inflammatory, antioxidant, immunomodulatory, and hypoglycemic properties [132,133]. Several studies have shown that GLP exerts potent anti-neuroinflammatory properties in a range of diseases [134,135]. GLP administration has been reported to significantly alleviate the inflammatory process by hindering the secretion of pro-inflammatory cytokines while promoting the secretion of anti-inflammatory cytokines [136,137]. In addition, GLP administration promoted microglia polarization from M1 to M2 phenotype, inhibited the JAK2/STAT3 signaling pathway to retinal ischemia–reperfusion (RIR), and had neuroprotective effects, making it a promising candidate in the field of glaucoma [138]. Qiu et al., using a formula composed of 13 herbs such as *Ganoderma lucidum* and *Lycium barbarum*, can alleviate inflammation by regulating MAPK and PI3K-Akt signaling pathways, as well as downstream iNOS and TNF-α, thereby improving retinal blood supply and vascular obstruction [139].

### 5.3. Salvia miltiorrhiza

*Salvia miltiorrhiza* is a traditional herb used in many Asian countries; it has been widely used to improve blood circulation and dissolve blood stasis [140,141]. There is growing evidence that *Salvia miltiorrhiza* exhibits potential anti-glaucoma effects in preclinical studies. Cryptotanshinone (CT) is a phenanthrenequinone obtained from the roots of *Salvia miltiorrhiza* with a unique phenanthrenequinone matric structure as well as biological activities [142]. It is capable of acting as an antibacterial and anti-inflammatory [143], anti-tumor [144], and cardiovascular protective agent [145]. The development of several retinal diseases is closely related to hypoxia. As a component of the traditional Chinese medicine *Salvia miltiorrhiza*, the effects of CT on retinal cells under hypoxic conditions are unknown [146]. A report in 2021 indicated that under hypoxic conditions, CT demonstrated its value as a therapeutic agent for the treatment of hypoxic ophthalmopathies by exerting apoptotic and inflammatory effects on protecting retinal pigment epithelium (RPE) cells from hypoxic conditions [147]. In addition, *Salvia miltiorrhiza* strengthens retinal tissue, which is particularly important for glaucoma patients, as changes in the retinal microenvironment are one of the important factors in RGC changes. Salvianic acid A, a unique water-soluble active ingredient of *Salvia miltiorrhiza*, contains relatively simple phenolic acids with antioxidant activity. It has been experimentally verified that Salvianic acid A can exert functional visual protective effects in vivo via dopamine D1 receptors [148].

### 5.4. Scutellaria baicalensis

The dried root of *Scutellaria baicalensis* is used as a medicine [149], and it has been shown to inhibit inflammation [150], oxidative stress [151], inhibit tumor growth [152], and protect nerves [153]. In addition, baicalin can reduce serum cholesterol, capillary permeability, and blood pressure [154]. Thus, the multiple pharmacological properties of Scutellaria baicalensis suggest its potential role in the experimental management of ocular diseases such as glaucoma, diabetic retinopathy [155], age-related macular degeneration [156], and cataracts [157]. *Scutellaria baicalensis* extract can dose-dependently exhibit protective effects against oxidative stress-induced RGC-5 damage [158].

Baicalein is a natural flavonoid reported to have a wide range of anti-inflammatory and neuroprotective bioactivities [159]. From the total constituents of five Chinese medicines (*Erigeron breviscapus*, *Salvia miltiorrhiza*, *Lycium barbarum*, *Crocus sativus* and Ginkgo biloba leaves), 143 active ingredients were screened. It was found that the use of baicalein from *Scutellaria baicalensis* extract significantly reduced IOP in a rat model of chronic high IOP. It protects the structure of retina and optic nerve, reduces apoptosis of RGCs, and effectively improves glaucoma symptoms [160]. Baicalein has also been found to improve macular edema caused by glaucoma [161]. A 2021 study showed that baicalein, another component of *Scutellaria baicalensis*, inhibited NMDA-induced apoptosis, autophagy, and oxidative stress in RGCs by activating PI3K/AKT signaling in vitro. In vivo studies have shown that baicalin activates PI3K/AKT signaling to inhibit autophagy, thereby attenuating pathological changes in the retinal tissue of mice with glaucoma [162]. Retinal vein occlusion (RVO) is a disorder of retinal circulation caused by narrowing of the retinal arteriovenous lumen and restricted diastolic function or vascular thromboembolism, which can result in neovascular glaucoma when RVO continues to develop [163]. Qin et al. found that baicalin could enhance the anti-apoptotic, anti-inflammatory, and antioxidant effects of ARPE-19 cells by inhibiting the HIF-1α/VEGFA axis in ex vivo experiments and improve BRVO in rats, which has therapeutic potential for glaucoma [161].

### 5.5. Silybum marianum

*Silybum marianum* has been found to have multiple therapeutic effects in the treatment of ocular surface diseases, cataracts [164], glaucoma, and uveitis [165]. Its pharmacological activities related to ocular diseases include anti-inflammatory, antioxidant, and antibacterial activities [166]. It also has anti-vascular endothelial growth factor (VEGF) and aldose reductase inhibitor properties, which may be beneficial for the treatment of certain eye diseases [164]. *Silybum marianum* contains several active ingredients; one of the main bioactive ingredients studied is Silibinin, which is made up of natural polyphenolic flavonoids [167]. It has been found to interfere with TGF-β1/Smad signaling in fibroblasts through possible pathosylation. These properties make milk thistle an attractive candidate for the treatment of various diseases, including glaucoma. Studies have shown that Silibinin attenuates TGF-β2-induced fibrotic changes in human trabecular reticulum cells by targeting the JAK2/STAT3 and PI3K/AKT signaling pathways. These findings suggest that Silibinin may serve as a potential intraocular pressure reducer by mitigating fibrotic changes in the trabecular meshwork tissues of patients with POAG [168]. Therefore, it is thought that Silibinin may play a role in the onset of neuropathy. Studies have shown that Silibinin can directly inhibit blue-induced acute RGC apoptosis and inflammation by activating the MEK/ERK/CREB pathway in vitro [169].

### 5.6. Astragalus membranaceus

Natural compounds derived from medicinal plants are becoming an important source of new drug development because they have the least side effects [170]. Astragaloside IV is a novel saponin isolated from the root of *Astragalus membranaceus*, a commonly used Chinese herbal medicine. It has been shown to have multiple therapeutic effects and a good safety profile compared to other natural products, making it a promising lead compound for drug discovery [171]. POAG is the most common type of glaucoma, accounting for nearly 74% of all glaucoma cases [172]. Elevated IOP was the only modifiable risk factor associated with POAG [173]. Astragaloside IV has been shown to reduce TGFβ2-induced extracellular matrix deposition and ER stress-related intraocular hypertension [174]. Additionally, it protects retinal cells from iron overload-induced ferroptosis by activating the Nrf2 signaling pathway [175]. The neuroprotective potential of *Astragalus membranaceus* polysaccharide, a water-soluble heteropolysaccharide, has been demonstrated in models of NMDA-induced retinal neurodegeneration. Inhibition of the NF-κB signaling pathway protects the retina from NMDA-induced damage and reduces the adverse effect of NMDA on the projection of RGC to the visual center as a potential novel therapeutic agent for the treatment of retinal diseases [114].

### 5.7. Ginkgo biloba

*Ginkgo biloba* is widely cultivated in Asian cultures, especially in China and Japan [176]. Its seeds are used for food and medicinal purposes to treat a variety of medical conditions and symptoms [177]. One of the most studied is ginkgo biloba. *Ginkgo biloba* extract (GBE) is mainly composed of flavonoids and terpenoids [178,179], which has the potential to widely solve the damage of glaucoma at the molecular level. Ginkgolide B (GB) is a unique diterpene lactone from *Ginkgo biloba* tree. It is one of the main active components in *Ginkgo biloba* extract and is known for its antagonistic effect on platelet-activating factor receptors [180]. However, recent studies have shown that GB in rat glaucoma models can counteract apoptosis in glaucoma models by inhibiting mitochondrial dysfunction, and the mitochondrial permeability transition pore mPTP is targeted as a potential intervention point [111]. In a 2022 study based on network pharmacology to predict the protective mechanism of ginkgo biloba against damaged retinal ganglion cells, the gene function of *Ginkgo biloba* that antagonizes RGC apoptosis was confirmed [181]. Additionally, *Ginkgo biloba* is a medicinal plant with excellent antioxidant properties whose effects have been demonstrated in a variety of degenerative processes, including retinal diseases associated with neurodegeneration [182]. Increased eye blood flow after the administration of Ginkgo biloba extract contributes to its potential anti-glaucoma effects shown in preclinical studies [183]. Although preclinical studies suggest that ginkgo extract may exert anti-glaucoma potential through mechanisms such as increasing ocular blood flow, antioxidation, and neuroprotection, the current clinical evidence is inconsistent. A recent systematic review [184] that comprehensively analyzes existing randomized controlled trials indicates that there is currently insufficient evidence to support the clinical efficacy of ginkgo extract for glaucoma patients. Therefore, the application of ginkgo extract in glaucoma treatment is still in the exploration stage, and larger-scale, multi-center clinical studies are needed for verification in the future.

### 5.8. Panax ginseng

*Panax ginseng* is the dried roots and rhizomes of Araliaceae plants, which have a variety of pharmacological active ingredients, mainly including ginsenosides, polysaccharides, volatile oils, amino acids, trace elements, and so on [185]. Among them, ginsenoside Rg1 is the main active ingredient of *Panax ginseng*, which has a variety of pharmacological effects such as antioxidant, anti-inflammatory, immunomodulatory, neuroprotective, etc. [186]. *Panax ginseng* itself does not directly act on atrial aquifer production or efflux pathway. However, its anti-inflammatory and antioxidant effects may help to reduce swelling and congestion of ocular tissues, thus indirectly affecting intraocular pressure. In animal models, ginseng extract has been shown to reduce optic nerve damage and promote neuronal regeneration and repair. For example, it has been shown that ginsenoside Rg1 was able to significantly improve levels of visual oxidative stress, intraocular pressure, and ganglion cell damage in a rabbit model of optic nerve damage with glaucoma [187]. The effect of ginsenoside Rg1 on the growth of retinal ganglion cells is poorly understood. One study from 2024 demonstrated that ginsenoside Rg1 promotes neurite growth of RGCs through the cAMP/PKA/CREB pathway, laying the foundation for clinical application in optic nerve diseases [113].

### 5.9. Crocus sativus

In recent years, an increasing number of preclinical studies have suggested that *Crocus sativus*, a traditional Chinese herb, may exert potential anti-glaucoma effects. Crocetin is a bioactive constituent of saffron derived from the stigma of *Crocus sativus*, family Iridaceae. Crocetin, as a chemical carotenoid derivative, has anti-inflammatory, antioxidant, and neuroprotective effects [188]. However, the protective effect of Crocetin on glaucoma and its mechanism is not clear. Studies have shown that Crocetin has neuroprotective and anti-inflammatory effects on retinal neurons in rats with glaucoma [189]. Microglial activation is a marker of neuroinflammation in the central nervous system [190]. Microglia, as immunomodulatory resident cells, may be able to initiate immune responses during neurodegeneration in glaucoma [83]. A 2019 report revealed the neuroprotective and anti-inflammatory effects of 3% hydrophilic Saffron Extract (dried stigmas of Crocus sativus) in a mouse model of unilateral laser-induced OHT. It partially reversed the downregulation of P2RY12 by reducing the number of OHT and contralilar microglia and the morphological signs of activation. In addition, the extract prevented RGC death in OHT models and reduced neuroinflammation associated with elevated IOP [191].

### 5.10. Resveratrol

Resveratrol is a non-flavonoid polyphenol compound with a wide range of biological activities [192]. Because of its potential antioxidant, anti-inflammatory, anticancer, and cardiovascular protective effects, it has attracted much attention [193]. Resveratrol is found in natural plants and fruits such as *Vitis vinifera*, *Senna obtusifolia* and *Rheum palmatum*. Resveratrol acts as a neuroprotectant that protects the optic nerve through a variety of mechanisms. Preclinical studies have shown that Resveratrol has good results in improving retinal ischemic injury in animal models of glaucoma. Resveratrol can significantly inhibit the upregulation of apoptosis-related protein Bax and lysed Caspase3 and increase the expression of Bcl2. In addition, resveratrol administration significantly inhibited the upregulation of HIF-1a/VEGF and p38/p53 pathways induced by retinal injury, while activating the downregulation of PI3K/Akt pathways. It can improve retinal function after retinal ischemic injury [194]. In addition, the antioxidant and anti-inflammatory properties of resveratrol have shown beneficial effects in TM cells. Studies have shown that resveratrol can increase NO production in TM cells in vitro, suggesting a potential role in modulating intraocular pressure, a key factor in the management of POAG [195].

**Table 2:** Summary of the biological characteristics of the chemical components extracted from the foodborne components.

Name	Components	Drug Administration	Biological Characteristics	Model	Disease Type	Major Studies	**Mechanisms**	**References**
*Lycium barbarum*	Polysaccharides		Antioxidant; anti-inflammatory; immunomodulatory; antiaging agent; neuronal responses	SD rats	AOH	To investigate the post-treatment neuronal rescue effects of LBP	Protects against secondary degeneration of RGCs; improves retinal function	[125]
	Polysaccharides	10 mg/kg	_	SD rats	OHT	To investigate the neuroprotective effects	Preserved RGC density and retinal functions	[126]
	Water-soluble polysaccharides from rhamnose, fructose, arabinose, galactose and galacturonic acid with molecular weights of 10–2300 kDa.	1 mg/kg	_	RGC-5 cells	CoCl_2_-induced oxidative damage model	To investigate RGC from CoCl2-induced apoptosis by mitochondrial membrane potential	Prevent RGC-5 cell apoptosis and lower ROS levels	[128]
	Ethanolic extracts	0.5 mg/mL	_	PC12 cells	Glaucoma	To investigate extract modulate extracellular matrix proteins	Inhibits MMP-9 expression; activates caveolin-1-dependent pathway	[127]
*Ganoderma lucidum*	Polysaccharides	50 and 100 mm	Anti-inflammatory; antioxidant; immunomodulatory; hypoglycemic	SD rats	RIR	To investigate the effect of GLP on retinal ischemia–reperfusion injury	Regulate JAK2/STAT3 pathway	[138]
	Aqueous extract	70 mg/kg	_	_	Retinal vein occlusion	To investigate the substance basis and the complex pharmacological activity of SQWMG on RVO treatment	MAPK and PI3K-Akt signaling pathways	[139]
*Salvia miltiorrhiza*	Cryptotanshinone 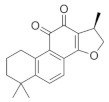	--	Blood circulation; removing blood stasis; reducing atherosclerosis	ARPE-19 cells	CoCl_2_-induced hypoxia model	To investigate how CT exerted its protective effects on RPE cells under hypoxic conditions induced by CoCl_2_.	Anti-inflammatory, anti-apoptotic and anti-VEGF properties	[147]
	Salvianic acid A 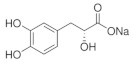	10 and 20 µM	_	ICR	Models of light-induced retinal damage	To investigate the protective effect of Salvianic acid A on retinal tissue and functional vision in vivo	The dopamine D1 receptors enhancement pathway	[148]
*Scutellaria baicalensis*	Baicalein 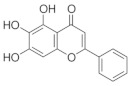	100 mg/kg	Anti-inflammatory; antioxidant; protect nerves; anti-tumor	SD rats	COH	To investigate the mechanisms and targets of baicalein in the glaucoma	Reduction in RGC apoptosis by upregulating the expression of the anti-apoptotic protein BCL-2	[160]
	Baicalin 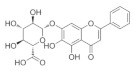	50 mg/kg	_	RGC-5 cells	NMDA-induced glaucoma	To investigate the effects of baicalin on RGC damage	Regulates PI3K/AKT signaling in vitro and in vivo	[162]
	Baicalin 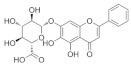	--	_	ARPE-19 cells/SD rats	CoCl_2_-induced model/BRVO rat model	To investigate the effects of baicalin on BRVO	Regulates the HIF-1α/VEGFA axis	[161]
*Silybum marianum*	Silibinin 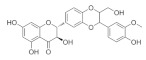	50 μg/mL	Anti-inflammatory; antioxidant; antibacterial	HTM cells	TGF-β2-induced model	To investigate changes in fibrosis induced in HTM cells treated with TGF-β2	Regulates JAK2/STAT3 and PI3K/AKT signaling pathways	[168]
	Silibinin 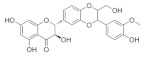	--	_	RGC-5 cells	Blue-induced acute model	To investigate the effect of Silibinin on blue light-induced acute RGC injury	Regulates MEK/ERK/CREB	[169]
*Astragalus membranaceus*	Astragaloside IV 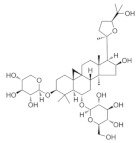	50 and 100 μM	Antifibrotic	TM cells/C57BL/6J mice	TGFβ2-induced model	To investigate the effect of Astragaloside IV on glaucomatous TM fibrosis, ER stress and ocular hypertension	Regulates NF-κB signaling pathways and MMPs	[174]
	Astragaloside IV 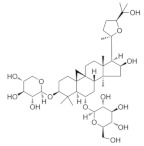	1 mM	_	ARPE-19 cells	Ferric ammonium citrate model	To investigate the protective mechanism of Astragaloside IV against iron overload-induced photoreceptor and retinal pigment epithelial cell death	Regulates Nrf2 signaling pathways	[175]
	Polysaccharides	--	Immunomodulatory; anti-inflammatory; cardioprotective properties	C57BL/6J mice	NMDA-induced model	To investigate the neuroprotective effect of polysaccharide on retinal cells in NMDA-induced retinal injury	Regulates NF-κB signaling pathways	[114]
*Ginkgo biloba*	Ginkgolide B (GB) 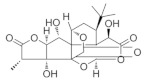	--	Anti-apoptotic	RPC cells/SD rats	Acute primary angle-closure glaucoma	To investigate the effectiveness of GB in combating cell death caused by glaucoma	Alleviates mitochondrial damage and apoptosis of RPC and retinal nerve fiber layer cells	[111]
	*Ginkgo biloba* extract	--	Anti-inflammatory; antioxidant	RGC-5 cells	EGb 761 and H_2_O_2_ model	To investigate the protective mechanism of Ginkgo biloba extract on damaged RGCs	By reducing the ratio of Bax/Bcl-2	[181]
*Panax ginseng*	Ginsenoside Rg1 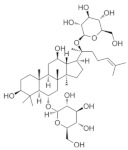	--	Anti-inflammatory; antioxidant	Rabbits	Ultrasound microbubbles destruction model	To investigate the treatment of glaucomatous optic nerve injury by ginsenoside Rg1 mediated by ultrasonic targeted microvesicle destruction	Reduce oxidative stress levels and relieve eye pressure and ganglion cell damage.	[187]
	Ginsenoside Rg1 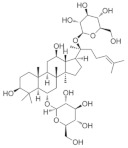	0.2 mL/kg	Anti-inflammatory; antioxidative stress; anti-apoptosis;	RGC-5 cells/ICR	_	To investigate the effect of ginsenoside Rg1 on neurite growth of RGCs	Regulates cAMP/PKA/CREB signaling pathways	[113]
*Crocus sativus*	Crocetin 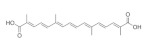	--	Anti-inflammatory; antioxidant; neuroprotective	SD rats	Glaucoma	To investigate the neuroprotective effect of *Crocus sativusl* on glaucoma model	Regulates PI3K/Akt/NF-κB signaling pathways	[189]
	Hydrophilic Saffron Extract	--	_	Male albino mice/Iba-1 + Cells	OHT	To investigate the neuroprotective and anti-inflammatory effects of hydrophilic Saffron Extract in a glaucoma model	Reduces neuroinflammation and RGC death	[191]
Resveratrol	Resveratrol 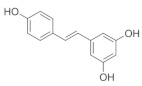	--	Antioxidant; anti-inflammatory; anticancer; cardiovascular	C57BL/6 J mice	A retinal ischemia–reperfusion injury model	To investigate the multiple pathways through which resveratrol attenuates the loss of RGCs in a mouse model of retinal ischemic injury	Reduces HIF-1a/VEGF, p38/p53 and PI3K/Akt	[194]
	Resveratrol 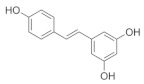	0.1, 1, 10 and 100 µM	_	GTM-3 cells	Glaucoma	To investigate the effect of resveratrol on inflammatory biomarkers of human trabecular reticulum cells in glaucomatous patients	The expression of eNOS was increased and the level of iNOS was decreased	[195]

### 5.11. Other Foodborne Bioactive Components

In addition to the ten commonly used foodborne components mentioned above, dietary supplements commonly used in daily life have been reported in glaucoma studies.

Green Tea: A randomized, double-blind, parallel-group clinical study involving 43 young healthy volunteers showed a reduction in IOP after oral administration of green tea and epigallocatechin gallate, compared with placebo [196]. However, given the study’s limited sample size and the exclusive inclusion of young participants without elevated IOP or glaucoma risk factors, these findings cannot be directly extrapolated to older adults or individuals with glaucoma. Further research in targeted populations is needed to determine whether green tea consumption may offer benefits for individuals at risk of glaucoma.

French maritime pine bark/bilberry fruit: The development of supplements with reduced IOP has broad market prospects. A recent study reported an oral supplement made from a mixture of French maritime pine bark and bilberry fruit in a ratio of 40 mg:90 mg. The results showed that supplement administration could reduce IOP in subjects and treat patients with POAG [197].

*Diospyros kaki*: Given the powerful free radical scavenging, antioxidant, and immunomodulatory properties of *Diospyros kaki*, Hong et al. investigated the pharmacological efficacy of *Diospyros kaki* in two glaucoma models, OHT and DBA/2 mice. The results showed that *Diospyros kaki* ethanol extract reduced the elevated IOP in two glaucoma mouse models. In particular, the finding of significant reductions in RGC loss and optic nerve damage supports the potential anti-glaucoma effects of *Diospyros kaki* shown in preclinical studies, suggesting its applicability as a health food [198].

Hesperidin: Hesperidin is a flavonoid found in citrus fruits that has a variety of biological activities. Its chemical structure is stable and it is easy to extract and purify, so it is widely used in food, healthcare products, and drugs. Hesperidin has been reported to have a certain antihypertensive effect on acute and chronic glaucoma rats, reducing glutamate levels and glutathione levels in aqueous humor. Taking all of this data together, it shows that Hesperidin supplements are effective against glaucoma in experimental rats [199].

## 6. Toxicity and Side Effects

Foodborne bioactive components are promising candidates for further study due to their properties of reducing intraocular pressure, ameliorating optic nerve damage, antioxidation, inhibiting the inflammatory response of ocular tissue, and reducing glutamate neurotoxicity. However, at present, the active components of the ingredients are complex and diverse, their specific pharmacological effects and mechanisms have not been fully defined, and there are still major obstacles in successfully transforming preclinical into routine clinical use. This may be attributed to differences in method of administration, dose, and duration [200].

Overall, more than a dozen of the foodborne bioactive components reviewed in this paper have a high safety profile at normal doses. However, some ingredients have also been shown to cause heart, liver, and kidney damage. White ginseng and red ginseng, for example, have no effect on the physiological structure and function of the rat myocardium, but they can induce arrhythmia and enhance the contractility of the heart, causing minor damage to the heart [201]. In addition, components may interact with other components. The combination of green tea and *Sambucus* RIL Ebulin triggered an important and unexpected synergistic toxic effect, with a fatality rate of more than 50% after 10 days [202].

Existing studies mainly focus on animal models and in vitro experiments, and clinical research data are limited, lacking long-term, large-scale, multi-center randomized controlled trials to verify their efficacy and safety. Studies of Crocus sativus have yet to determine the effective dose and long-term toxicity in vivo [203]. Although preclinical studies suggest that many plant-derived compounds have potential for treating glaucoma, the information regarding the toxicity and side effects of these compounds when used in the human body (especially for glaucoma patients) is currently extremely limited. Apart from a few ongoing clinical trials, current knowledge about the long-term medication safety, optimal dosing regimens, and potential drug interactions for the glaucoma population is still very limited.

## 7. Discussion

Mechanisms such as high intraocular pressure, oxidative stress, and neuroinflammatory microenvironment can activate relevant signaling pathways, leading to irreversible apoptosis of retinal neurons and axonal degeneration, thus accelerating the progression of glaucoma. This paper systematically describes the mechanisms responsible for the potential anti-glaucoma effects shown in preclinical studies of foodborne bioactive components (such as polysaccharides, flavonoids, etc.), involving targeted regulation of NF-κB, JAK2/STAT3, PI3K/AKT, MEK/ERK/CREB, cAMP/PKA/CREB, and other key pathways. Studies have shown that *Lycium barbarum* (polysaccharides, ethanol extracts, etc.), *Astragalus membranaceus,* and other ingredients can significantly reduce RGC damage induced by oxidative stress, inhibit RGC inflammation, and thus reduce the release of retinal inflammatory factors (IL-1β, IL-18). Some ingredients are able to protect RGCs from the degenerative changes that increase eye pressure and RGC death. In addition, some plant extracts (such as resveratrol) have been found to improve retinal function by regulating the PI3K/Akt pathway, indirectly alleviating RGC damage.

Although foodborne bioactive components have shown protective effects on the optic nerve in animal models, their clinical application still faces challenges. For example, component interactions such as green tea may affect the synergistic toxicity of drugs in vivo, and the drug dosage needs to be clearly specified. At the same time, most existing studies focus on in vitro cell models or animal glaucoma models, lacking validation of the clinical retinal microenvironment and human clinical data. There are numerous challenges in transitioning from preclinical evidence to clinical application. The issue of bioavailability is a major concern. Most foodborne components are poorly water-soluble and metabolize rapidly, making it difficult for them to reach an effective concentration in the retina.

In recent years, the development of advanced delivery systems has provided new ideas for breaking through this bottleneck. New delivery carriers such as nanoparticles, liposomes, micelles, dendritic macromolecules, and hydrogels can significantly improve the intraocular bioavailability of active ingredients by enhancing corneal permeability, prolonging the retention time at the ocular surface, and achieving targeted release. Future research should combine advanced delivery technologies with the screening of food-derived active ingredients, and based on the identification of active substances, solve the problems of their in vivo delivery through formulation techniques, thereby accelerating the clinical translation process of these substances.

The multi-target synergy is different from the logic of drug development. Whether short-term indicators can be transformed into long-term visual function protection requires a huge amount of long-term trial verification. The course of glaucoma lasts for several decades, and the existing models are unable to simulate its chronic progression, which is also an important reason for the low success rate of clinical translation. Meanwhile, most existing studies have mixed the crude extracts with the individual compounds. There are fundamental differences in mechanism and utilization between the two, but due to a lack of sufficient evidence, it is currently difficult to conduct a strict classification and discussion. Although food-derived active ingredients show great potential in the intervention of glaucoma, their clinical application must be based on a thorough safety assessment. Future research should focus on the chronic toxicity of long-term use, the safety of local ocular application, and the interactions with conventional anti-glaucoma drugs. A comprehensive evidence-based safety database should be established. At the same time, clinicians should be more vigilant about patients’ self-combination of dietary supplements, and strengthen the inquiry into medication history and risk communication in clinical practice. The lack of safety information regarding the toxicity and side effects of these compounds in the human body is one of the important bottlenecks restricting the clinical application of these compounds.

Future studies should focus on the optimization of blood–retinal barrier penetration of foodborne bioactive components and explore their potential in combination with existing IOP drugs. The analysis of the multi-target regulatory network of foodborne bioactive components on the pathogenesis pathway of glaucoma is expected to provide a new strategy for the development of neuroprotective anti-glaucoma therapy based on natural products. Future research should fully utilize cutting-edge technologies such as network pharmacology, systems biology and bioinformatics to break through the current limitations of single pathways and single targets, and comprehensively explain the molecular mechanism by which food-derived active components intervene in glaucoma. This will provide a more profound theoretical basis for precise nutritional intervention and clinical application transformation of food-derived functional components.

## 8. Conclusions

In conclusion, preclinical studies have revealed that various plant-derived foods, by regulating multiple signaling pathways such as NF-κB, PI3K/AKT, JAK2/STAT3, etc., exhibit potential anti-inflammatory, antioxidant, and neuroprotective effects, as well as the ability to lower intraocular pressure, against glaucoma. However, it is necessary to clearly point out that the current evidence mainly comes from in vitro experiments and animal models, and human clinical research is extremely limited. Moreover, the results of existing clinical trials are inconsistent. Currently, the long-term safety, optimal dosing regimen, and potential drug interactions of these compounds in glaucoma patients are still unknown, and the clinical data is limited.

## Figures and Tables

**Table 1:** Current glaucoma treatments.

Type of Treatment	Advantages	Limitations
Medical therapy	Easy to use; high security	Long-term medication; side effects
Laser therapies	Simple operation; good therapeutic effects	Not for all types of glaucoma; causing complications
Surgical therapies	Wide range of applications; little recurrence	High surgical risk; causes complications

## Data Availability

No new data were created or analyzed in this study.

## Abbreviations

AH, aqueous humor; AL, axial length; AC, anterior chamber; BRB, blood–retinal barrier; ASC, apoptosis-associated speck-like protein; AOH, acute ocular hypertensive; BRVO, branch retinal vein occlusion; BNDF, brain-derived neurotrophic factor; CNTF, ciliary neurotrophic factor; CB, ciliary body; COH, chronic ocular hypertension; DAMP, damage-associated molecular patterns; ECM, extracellular matrix; IOP, intraocular pressure; LC, Lamina Cribrosa; MMPs, metalloproteinases; mPTP, mitochondrial permeability transition pore; NMDA, N-methyl-D-aspartate; NMDAR, NMDA receptor; NTG, normal tonic glaucoma; NGF, nerve growth factor; NOS, nitric oxide synthase; NO, nitrogen monoxide; PC, posterior chamber; PACG, primary angle-closure glaucoma; POAG, primary open-angle glaucoma; RGC, retinal ganglion cell; RGCs, retinal ganglion cells; RPCs, retinal progenitor cells; ROS, reactive oxygen species; RIR, retinal ischemia–reperfusion; RPC, retinal progenitor; RVO, retinal vein occlusion; RPE, retinal pigment epithelium; SC, Schlemm’s canal; TMCs, trabecular meshwork cells; TM, trabecular meshwork; VEGF, vascular endothelial growth factor.
